# The Intriguing Role of Interleukin 13 in the Pathophysiology of Asthma

**DOI:** 10.3389/fphar.2019.01387

**Published:** 2019-12-06

**Authors:** Giancarlo Marone, Francescopaolo Granata, Valentina Pucino, Antonio Pecoraro, Enrico Heffler, Stefania Loffredo, Guy W. Scadding, Gilda Varricchi

**Affiliations:** ^1^Department of Public Health, University of Naples Federico II, Naples, Italy; ^2^Azienda Ospedaliera Ospedali dei Colli, Monaldi Hospital Pharmacy, Naples, Italy; ^3^Department of Translational Medical Sciences and Center for Basic and Clinical Immunology Research (CISI), University of Naples Federico II, Naples, Italy; ^4^WAO Center of Excellence, University of Naples Federico II, Naples, Italy; ^5^College of Medical and Dental Sciences, Institute of Inflammation and Ageing, University of Birmingham, Birmingham, United Kingdom; ^6^Immunodeficiency Centre for Wales, University Hospital of Wales, Cardiff, United Kingdom; ^7^Personalized Medicine, Asthma, and Allergy, Humanitas Clinical and Research Center, IRCCS, Milan, Italy; ^8^Department of Biomedical Sciences, Humanitas University, Milan, Italy; ^9^Institute of Experimental Endocrinology and Oncology “G. Salvatore” (IEOS), National Research Council (CNR), Naples, Italy; ^10^Allergy and Clinical Immunology, Imperial College, National Heart and Lung Institute, London, United Kingdom

**Keywords:** asthma, biologics, chronic rhinosinusitis, interleukin 4, interleukin 13, nasal polyposis, tralokinumab

## Abstract

Approximately 5–10% of asthmatic patients worldwide suffer from severe asthma. Experimental and clinical studies have demonstrated that IL-13 is an important cytokine in chronic airways inflammation. IL-13 is involved in Th2 inflammation and has been identified as a possible therapeutic target in the treatment of asthma. Two different human monoclonal antibodies (mAbs) anti-IL-13 (tralokinumab and lebrikizumab) block binding and signaling of IL-13 to its receptors, IL-13Rα1 and IL-13Rα2. Several randomized, double-blind, placebo-controlled multicenter studies have evaluated the safety and efficacy of tralokinumab and lebrikizumab in the treatment of adult patients with severe asthma, but all have failed to meet their primary endpoints. No serious adverse events related to the treatment with these anti-IL-13 mAbs have been reported in these studies. These negative clinical results contrast with positive findings from blocking IL-13 signaling in experimental models of asthma, raising doubts about the transferrable value of some models. Interestingly, dupilumab, a mAb which blocks both IL-4 and IL-13 signaling reduces exacerbation rates and improves lung function in severe asthmatics. These results suggest that IL-4 and IL-13 share some, but not all functional activities in airway inflammation. Tralokinumab might show efficacy in a highly selected cohort of asthmatics characterized by overexpression of IL-13.

## Introduction

Bronchial asthma is a chronic inflammatory disorder of the airways characterized by reversible airflow obstruction, bronchial hyperreactivity (BHR), mucus overproduction, angiogenesis, and airway remodeling (Detoraki et al., 2010; Holgate et al., 2015). Asthma is a common disorder resulting in substantial morbidity, healthcare expenditure, and death (Lang and Polansky 1994; Lange et al., 1996). Worldwide, up to 300 million people are affected by asthma, making it one of the most common chronic diseases (World Health Organization. Global surveillance, prevention, and control of chronic respiratory diseases. http://www.who.int/gard/publications/GARD/2007) and approximately 250,000 people die from asthma each year (Lozano et al., 2012). In the majority of patients, asthma can be controlled by combinations of inhaled glucocorticoids (ICS), short- or long-acting ß_2_-adrenergic agonists (LABA), long-acting muscarinic antagonists, and leukotriene receptor antagonists, according to the Global Initiative for Asthma (GINA) guidelines (GINA http://www.ginasthma.org/pdf). However, in approximately 15% of patients, the disease is refractory to conventional treatments (Chachi et al., 2013; Chachi et al., 2017) and results in hospital admissions due to severe exarcerbations (Sekiya et al., 2016; Kerkhof et al., 2018).

As with many chronic inflammatory diseases, clinicians now realize that the traditional classification of asthma has been an oversimplification (Marone et al., 2005). Different asthma phenotypes, each with distinct pathophysiology, are now being defined as asthma endotypes (Wenzel 2012; Fahy 2015; Hinks et al., 2016; Terl et al., 2017). The heterogeneity of different forms of asthma reflects the involvement of different immune cell populations and inflammatory mediators (Bagnasco et al., 2017; Varricchi et al., 2017; Marone et al., 2019). Asthma can be classified according to two major endotypes: “T2-high” asthma is characterized by increased levels of type 2 inflammation mainly mediated by mast cells, eosinophils, basophils, T-helper 2 cells, group 2 innate lymphoid cells (ILC2s), and immunoglobulin E (IgE)-producing B cells (Fahy, 2015). Patients with T2-high asthma have eosinophilia and other signs of type 2 inflammation including high levels of IL-4 and IL-13 (Fahy, 2015; Kaur and Chupp, 2019). Increased blood and sputum levels of eosinophils, serum IgE, and the fraction of exhaled nitric oxide (FeNO) have been associated with the mechanisms of T2-high asthma (Gandhi et al., 2017; Robinson et al., 2017). Another feature is the high expression of the prostaglandin D_2_ receptor chemoattractant receptor-homologous molecule (CRTH_2_) on Th2 lymphocytes (Cosmi et al., 2000; Marone et al., 2019). “T2-low” asthma is less well characterized and may include several different endotypes (Wenzel, 2012; Choy et al., 2015; Fahy, 2015; Pepper et al., 2017). It has been suggested that Th1 and Th17 pathways and neurogenic inflammation may be involved (Ricciardolo et al., 2017; Samitas et al., 2017).

Levels of IgE, blood eosinophils and FeNO can be useful to guide the selection of monoclonal antibodies (mAbs) in the treatment of different forms of severe uncontrolled asthma. For example, several studies have demonstrated the safety and efficacy of mAbs blocking IL-5 (i.e., mepolizumab and reslizumab) for treatment of patients with severe eosinophilic asthma (Bel et al., 2014; Ortega et al., 2014; Bjermer et al., 2016; Khatri et al., 2019). Benralizumab, a mAb against IL-5Rα, expressed on the surface of human eosinophils (Varricchi et al., 2018a), is particularly effective in patients with severe asthma with high blood eosinophils (FitzGerald et al., 2016; FitzGerald et al., 2018). Because IgE and the high affinity receptor for IgE (FcεRI) play a central role in atopic asthma (Varricchi et al., 2018d; Borriello et al., 2019; Varricchi et al., 2019), a mAb anti-IgE (omalizumab) is indicated for the treatment of patients (aged ≥ 12 years) with moderate-to-severe uncontrolled allergic asthma (Samitas et al., 2015; Hew et al., 2016). Serum concentrations of IgE are used to guide this treatment.

IL-4 and IL-13 were among the first identified cytokines orchestrating Th2 inflammation (Macchia et al., 2015; McCormick and Heller 2015; Bagnasco et al., 2016). IL-4 and IL-13 are potent mediators of type 2 inflammation with both overlapping and distinct functions. Pascolizumab, a mAb selectively blocking IL-4, failed to produce positive effects (Hart et al., 2002). Two mAbs blocking IL-13 (i.e., anrukinzumab, lebrikizumab) have shown marginal effects in the treatment of asthmatic patients (Corren et al., 2011; Gauvreau et al., 2011; Hanania et al., 2015; Bagnasco et al., 2016; Hanania et al., 2016). Another mAb anti-IL-13 (i.e., tralokinumab, AstraZeneca) failed to reduce asthma exacerbation rate in severe uncontrolled asthmatics (Piper et al., 2013; Brightling et al., 2015; Panettieri et al., 2018; Russell et al., 2018). Considering the individual relevance of IL-4 and IL-13 in the pathogenesis of asthma, these results are surprising and intriguing. A simplistic explanation is that individual blockade of IL-4 or IL-13 is insufficient to inhibit the complex orchestration of allergic inflammation and clinical consequences in severe asthma. This hypothesis is indirectly supported by the efficacy of dupilumab, which inhibits both IL-4 and IL-13 signaling mediated by IL-4Rα, in patients with severe uncontrolled asthma (Castro et al., 2018; Rabe et al., 2018).

In this review we analyze the biological and immunological effects of IL-13 in the context of experimental models of asthma and in asthmatic patients. Despite promising findings in several experimental models of allergic inflammation, the results of multicenter studies evaluating the efficacy of anti-IL-13 mAbs in patients with asthma were surprisingly negative. Possible explanations of these discrepancies are discussed.

### Biological and Immunological Effects of Interleukin 13

IL-13 is a pleiotropic cytokine originally cloned from activated human T-lymphocytes (Minty et al., 1993). The human *IL13* gene is located on chromosome 5q31-33 in the cluster of genes encoding IL-4, IL-3, IL-5, IL-9, and granulocyte-macrophage colony-stimulating factor (GM-CSF). The gene encoding IL-13 is upstream of the *IL4* gene, leading to the speculation that these genes arose as a duplication event during evolution. However, IL-13 has only 25% homology with IL-4 thus explaining why these cytokines share some, but not all functional properties. IL-13 can be produced by stimulated Th2 cells (de Vries 1998), B lymphocytes (Hajoui et al., 2004), CD8^+^ cells (Dakhama et al., 2013), type 2 ILCs (Jia et al., 2016), alveolar macrophages (Hancock et al., 1998), human mast cells (Fushimi et al., 1998), and basophils (Ochensberger et al., 1996; Redrup et al., 1998; Borriello et al., 2015).

**Figure 1:**
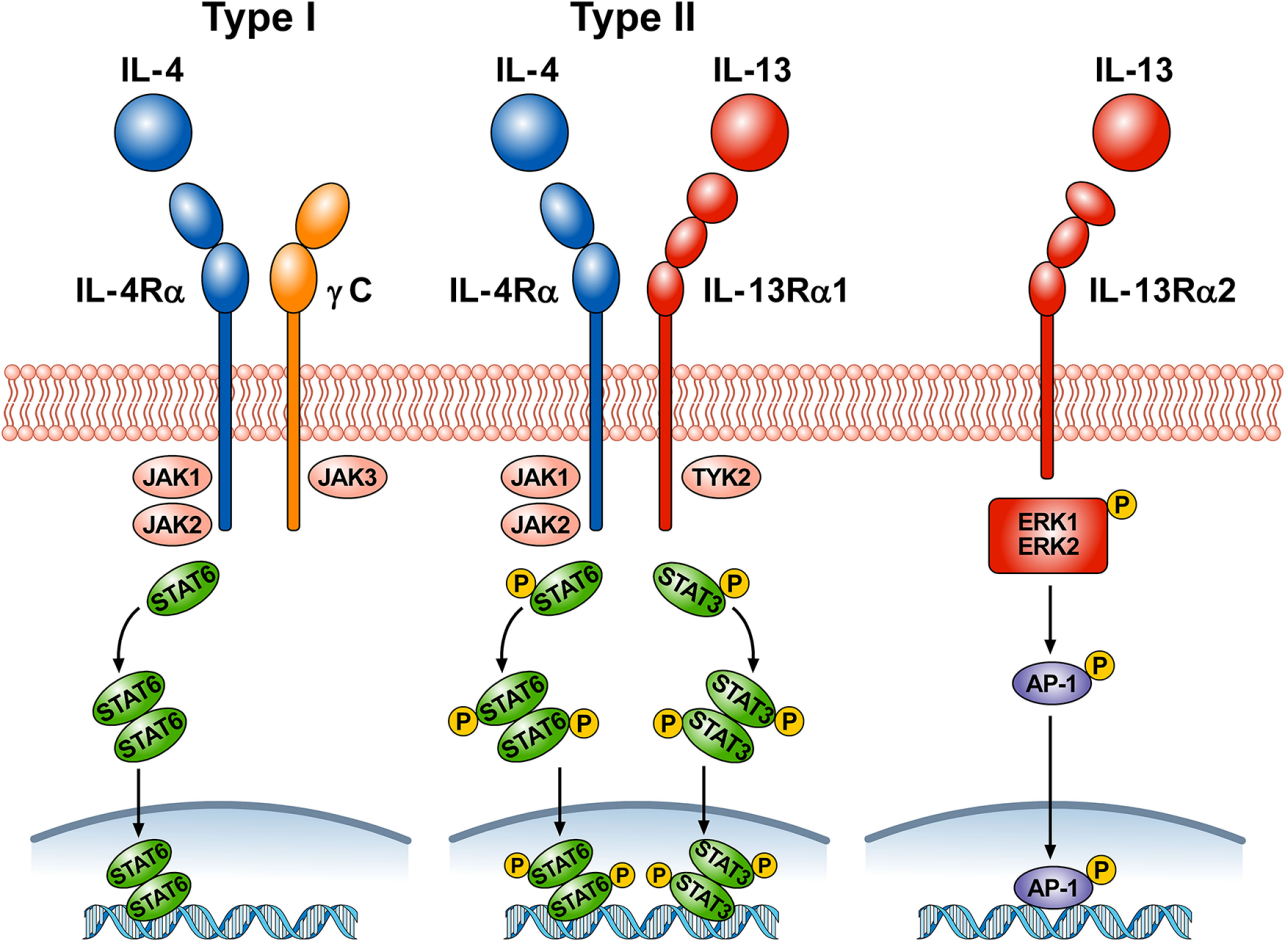
Schematic representation of the three receptors that bind IL-4, IL-13, or both. Type I receptor is composed of the IL-4Rα subunit complexed with common γc. This receptor, expressed on hematopoietic cells, binds to IL-4. Ligand binding by type I receptor complex leads to activation of Janus family kinases (JAK1, JAK2, and JAK3) and subsequent phosphorylation of signal transducer and activator transcription 6 (STAT6). Type II receptor consists of IL-4Rα complexed with IL-13Rα1 and is found in many non-hematopoietic cells (e.g., bronchial epithelial cells, smooth muscle cells, fibroblasts, keratinocytes). Ligand binding type II receptor complex leads to activation of JAK1, JAK2, and tyrosine kinase 2 (TYK2) and subsequent phosphorylation of STAT6 and STAT3. Activation of JAKs leads to the recruitment of STATs to the receptors, followed by STAT phosphorylation and dimerization. Activated STAT dimers translocate to the nucleus, bind specific DNA elements, and initiate activation of downstream genes. IL-4 signals through both type I and type II receptors, whereas IL-13 signals only through type II receptor. IL-13 also binds to a third IL-13Rα2 receptor whose functions are largely unknown. Under certain circumstances, IL-13 signaling through IL-13Rα2 results in phosphorylation of ERK1/2 in a STAT6-independent manner and the formation of the dimeric transcription factor AP-1. Phosporylated AP-1 translocates to the nucleus and bind to specific DNA elements.

**Figure 1** schematically illustrates the complex receptor system which mediates the signaling of IL-4 and IL-13. The IL-4Rα subunit is a component of both the type I and type II receptors. Type I receptors are composed of the IL-4Rα subunit complexed with common γ chain (γc); this receptor binds to IL-4 and is expressed on cells of hematopoietic stem cell origin. The type II receptor complex consists of IL-4Rα partnering with IL-13Rα1 and is found on many non-hematopoietic cells, such as bronchial epithelial cells, smooth muscle cells, fibroblasts, and keratinocytes (Akaiwa et al., 2001). IL-4 signals through both the type I and type II receptor complexes whereas IL-13 signals only through the type II complex, because IL-13 binds to IL-13Rα1, whereas IL-4 primarily binds to IL-4Rα (McKenzie et al., 1999). In addition, the two cytokines have different functions and signaling. IL-4Rα, γc, and IL-13Rα1 all contain proline rich regions that can bind the Janus kinases JAK1, JAK2, JAK3, and TYK2. In hematopoietic cells that express γc and the associated JAK3, IL-4 binding to type I receptor results in the activation of JAK1, JAK2, and JAK3 (Hershey, 2003; Bhattacharjee et al., 2013). IL-4 and IL-13 binding to type II receptor activate JAK1, JAK2, and TYK2. Activation of JAKs results in phosphorylation of cytoplasmic tyrosines leading to the recruitment of STAT6 to the receptor, followed by its phosphorylation and activation. The activation of STAT6 is the primary signaling event in the response to IL-4 or IL-13 (Cao et al., 2016). In certain experimental conditions STAT1 and STAT3 can also be activated by both IL-4 and IL-13 (Wang et al., 2004; Bhattacharjee et al., 2013; Pham et al., 2019). The cytoplasmic domain of human IL-13Rα1 contains two tyrosine residues, which might serve as docking sites for STAT3 (Hershey, 2003). Phosphorylated STAT6 and STAT3 monomers dimerize and then translocate to the nucleus, bind to specific DNA elements to regulate transcription (Bhattacharjee et al., 2013).

LaPorte and collaborators have examined in detail the molecular and structural basis of the IL-4/IL-13 receptor system (LaPorte et al., 2008). They demonstrated that IL-4 first binds to IL-4Rα to form a binary complex which then binds to the γc to form the functional ternary complex (type I receptor). IL-4 and IL-13 can also bind with high affinity to IL-4Rα and IL-13Rα1, respectively (type II receptor). The authors also compared the kinetics and potency of IL-4 and IL-13 signaling. IL-4 induced tyrosine phosphorylation of STAT6 more rapidly and more potently than IL-13. The latter observation was supported by previous experiments on cultured airway smooth muscle cells (Laporte et al., 2001). IL-4 is a central mediator for Th2 cell polarization, initiation of IgE synthesis, and recruitment of eosinophils (Chatila, 2004; Wynn, 2015). Although IL-13 has some redundancy in these effects, this cytokine has additional roles in mediating goblet cell hyperplasia, airway smooth muscle contractility, collagen deposition, and fibrosis (Gour and Wills-Karp 2015).

There is a distinct IL-13Rα2 subunit, to which only IL-13 binds (Chen et al., 2009). Initially this receptor was considered a decoy receptor (Ingram and Kraft, 2012) involved in removing IL-13 by internalization (Wood et al., 2003; Lupardus et al., 2010; Kasaian et al., 2011). Although the IL-13Rα2 lacks canonical JAK-STAT signaling activity (Kawakami et al., 2001), this hypothesis has come into question because several studies have shown that, under certain circumstances, IL-13Rα2 can mediate IL-13 signaling (Fichtner-Feigl et al., 2006; Fujisawa et al., 2009; He et al., 2013). In human airways, it was found that IL-13Rα2 is involved in IL-13 signaling through the transcription factor activator protein-1 (AP-1) to induce the activation of TGF-β (Fichtner-Feigl et al., 2006). Recently, it has been demonstrated that IL-13 induced phosphorylation of ERK1/2 and the downstream activation of AP-1-related genes in human nasal epithelial cells (Liu et al., 2018). The authors proposed that engagement of IL-13Rα2 by IL-13 activates mitogen-activated protein ERK1/2 pathway and downstream AP-1-related gene *C-JUN*.

**Figure 2:**
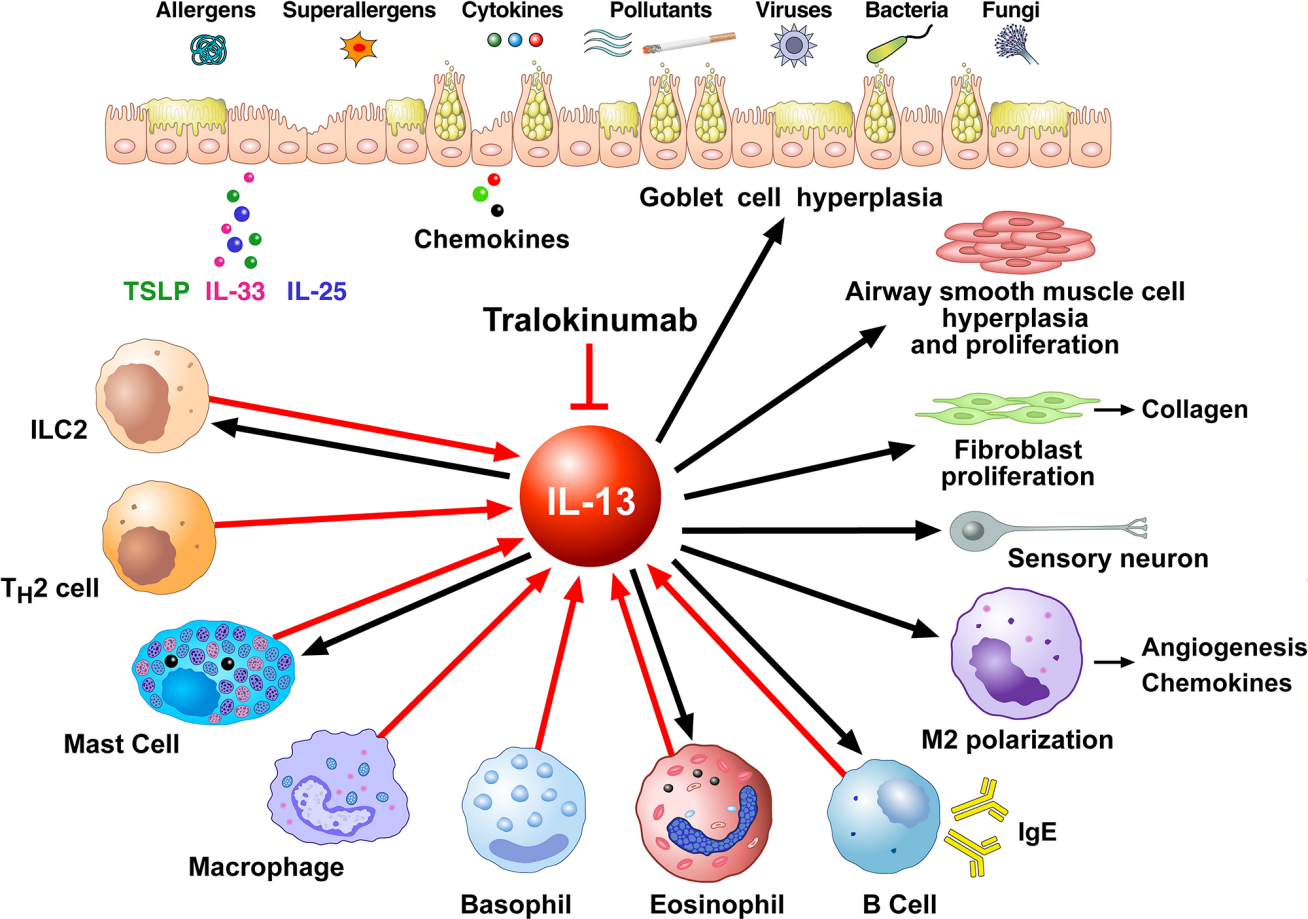
Schematic representation of the cellular sources of IL-13 (red arrows) and its effects of immune and structural cells in asthma (black arrows). Different stimuli (allergens, superallergens, pollutants, viral and bacterial products, etc.) activate epithelial cells which release several cytokines (e.g., thymic stromal lymphopoietin, IL-33, IL-25). These mediators activate a variety of immune cells (ILC2, Th2 cells, mast cells, macrophages, basophils, eosinophils, B cells) which produce several cytokines including IL-13. IL-13 modulates the functions of IL-13^+^ ILC2, mast cells, macrophages, eosinophils, and B cells. This cytokine induces goblet cell hyperplasia and mucus production, airway smooth muscle cell hyperplasia and proliferation, fibroblast activation and collagen deposition, macrophage polarization (M2) and B cell activation and immunoglobulin E production. These effects of IL-13 are mediated by the engagement of type II IL-13 receptor (IL-13Rα1/IL-4Rα expressed on these cells). IL-13, as well as IL-4, can activate sensory neurons through the engagement of type II receptor. Tralokinumab, an anti-IL-13 human IgG_4_ monoclonal antibody, binds IL-13, thus preventing it from binding to both IL-13Rα1 and IL-13Rα2 on target cells.

**Figure 2** shows schematically that IL-13 is produced by several immune cells and has many diverse functions on a wide variety of cell types relevant to the pathogenesis of allergic disorders. IL-13 can be produced by activated ILC2 (Shimokawa et al., 2017; Wallrapp et al., 2018), Th2 cells (Finkelman et al., 2004; Wynn, 2015), mast cells (Burd et al., 1995; Fushimi et al., 1998; Varricchi et al., 2019), macrophages (Hancock et al., 1998), basophils (Gibbs et al., 1996; Ochensberger et al., 1996; Redrup et al., 1998; Patella et al., 2000; Genovese et al., 2003), eosinophils (Schmid-Grendelmeier et al., 2002; Varricchi et al., 2018a), and B cells (Hajoui et al., 2004). In human B cells IL-13 has similar effects as IL-4, including promoting B cell proliferation and inducing class switching to IgE and IgG_4_ in combination with CD40/CD40L (Oettgen and Geha, 2001) and inducing expression of the low-affinity IgE receptor CD23 (Punnonen et al., 1993; Gould and Sutton, 2008). In macrophages IL-13 favors the M2 polarization (Martinez-Nunez et al., 2011; Bhattacharjee et al., 2013). IL-13 promotes survival, activation, and recruitment of eosinophils (Luttmann et al., 1996; Horie et al., 1997; Pope et al., 2001). In addition, IL-13 stimulates eosinophil trafficking from the peripheral blood to the site of inflammation by inducing the production of IL-5 and eosinophil chemokines such as eotaxins (Webb et al., 2000; Rosenberg et al., 2007) IL-13 promotes FcεRI expression and proliferation of human mast (Nilsson and Nilsson, 1995; Kaur et al., 2006).

IL-13 also has important effects on non-hematopoietic cells, including endothelial cells, smooth muscle cells, fibroblasts, epithelial cells, and sensory neurons. IL-13 is a potent inducer of vascular cell adhesion molecule-1 (VCAM-1) on endothelial cells, an important aspect in the recruitment of eosinophils (Bochner et al., 1995). In addition, IL-13 increases the expression of ß_1_ integrin and VCAM-1 on human lung fibroblasts (Doucet et al., 1998) and increases muscular contraction in response to acetylcholine (Laporte et al., 2001; Grunstein et al., 2002). Furthermore, IL-13 enhances proliferation and cholinergic-induced contraction of smooth muscle cells (Wills-Karp, 2001) and induces collagen synthesis in human fibroblasts contributing to airways remodeling. In epithelial cells, IL-13 is a potent inducer of eotaxin (Li et al., 1999). Moreover, IL-13 induces mucus overproduction and goblet cell metaplasia (Kuperman et al., 2002; Kondo et al., 2006). IL-13 induces vascular endothelial growth factors (VEGFs) (Corne et al., 2000) which are pro-angiogenic factors relevant in bronchial asthma (Detoraki et al., 2009; Detoraki et al., 2010; Varricchi et al., 2018b). Recently, it has been demonstrated that IL-13 and IL-4 directly activate mouse and human sensory neurons which express IL-13Rα1 and IL-4Rα (Oetjen et al., 2017).

### Interleukin 13 in Experimental Models of Asthma

Seminal studies in animal models of allergic asthma demonstrated that selective neutralization of IL-13 reduced airway hypersensitivity (AHR), bronchoalveolar lavage (BAL) eosinophils, and mucus overproduction (Grunig et al., 1998; Wills-Karp et al., 1998). Furthermore, IL-13 delivery to the airways caused all of these effects (Grunig et al., 1998; Wills-Karp et al., 1998). Overexpression of IL-13 in the lung of mice caused mucus hypersecretion, subepithelial fibrosis, eotaxin production, and eosinophilic infiltration (Zhu et al., 1999). Interestingly, mice with targeted deletion of IL-13 failed to develop allergen-induced AHR, despite the presence of eosinophilic pulmonary infiltration (Walter et al., 2001). Mice lacking STAT6 were protected from pulmonary effects of IL-13 (Kuperman et al., 2002). Importantly, reconstitution of STAT6 in epithelial cells only was sufficient for IL-13-induced AHR and mucus production in the absence of inflammation and fibrosis. Administration of anti-IL-13 in a mouse model of chronic asthma inhibited eosinophil recruitment in the airways, goblet cell hyperplasia, and subepithelial fibrosis, but only marginally inhibited AHR (Kumar et al., 2004). In another study, blockade of IL-13 with sIL-13Rα2-human IgG fusion protein inhibited AHR associated with brief allergen exposure, but did not modify AHR associated with chronic airway remodeling (Leigh et al., 2004).

An IL-13 vaccine prepared by inserting a murine IL-13 peptide into a viral carrier protein, induced sustained and intense anti-IL-13 IgG antibodies (Ma et al., 2007), associated with inhibition of ovalbumin (OVA)-induced acute airway allergic responses. In a more recent study, the same experimental approach suppressed BAL IL-13 concentration and eosinophils, subepithelial collagen deposition, and mucus hyperproduction (Ma et al., 2013). Interestingly, while IL-13 vaccine inhibited AHR development, it did not revert AHR. The latter findings suggest that IL-13 may be crucial in the development, but not in the maintenance of airway hyperesponsiveness.

*Il13ra2* gene silencing or blockade of IL-13Rα2 signaling led to marked downregulation of TGF-β1 production and collagen deposition in a model of lung fibrosis (Fichtner-Feigl et al., 2006). In IL-13Rα2-deficient mice, AHR and airway inflammation (i.e., mucus production and BAL eosinophils) were attenuated compared to wild type mice following house dust challenge (Chen et al., 2013). More recently, it was reported that, in IL-4Rα-deficient mice, IL-13, but not IL-4, was required for development of OVA-mediated AHR and goblet cell hyperplasia (Kirstein et al., 2016). Munitz and collaborators studying *Il13ra1* ^-/-^ mice found that IL-13Rα1 is critical for baseline IgE production, AHR, mucus production, and eotaxin production. By contrast, Th2 and IgE responses to antigen were IL-13Rα1-independent (Munitz et al., 2008).

In a novel mouse model of non-allergic asthma overexpression of the activator protein-1 (AP-1) subunit Fra2, caused airway inflammation with IL-13 overexpression, BAL eosinophilia, mucus hyperproduction, AHR, and peribronchial collagen deposition (Gungl et al., 2018). Administration of anti-IL-13 antibody markedly decreased STAT6 phosphorylation in the lung, BAL eosinophilia, and goblet cell hyperplasia. However, peribronchial collagen deposition and bronchial smooth muscle width were not affected by anti-IL-13 administration. The interesting results obtained in this experimental model may be more reflective of severe asthma which exhibit poor response to mAb anti-IL-13.

Genetic deletion of the IL-33 receptor in a mouse model of experimental asthma increased TSLP production, which stimulated the emergence of IL-13^+^ ILC2s and lung mast cells leading to airway hyperresponsiveness (Verma et al., 2018).

Collectively, the results derived from different experimental models indicate that IL-13 plays an important role in the development of several aspects of asthma. However, it is becoming evident that IL-13 neutralization is not sufficient to reverse certain aspects of airway inflammation once they are established.

### Interleukin 13 Expression in Asthmatic Patients

Increased concentration of IL-13 have been found in the blood (Alasandagutti et al., 2017), sputum, bronchial mucosa (Berry et al., 2004), and BAL fluid (Prieto et al., 2000) of asthmatic patients compared to healthy individuals. Increased IL-13 expression in asthma was confirmed by IL-13 mRNA overexpression (Truyen et al., 2006) and by *ex vivo* stimulation of sputum T cells (Boniface et al., 2003). Following allergen challenge, IL-13 is increased in BAL (Huang et al., 1995; Kroegel et al., 1996). *Ex vivo* BAL T cells express IL-13 mRNA (Bodey et al., 1999); expression is inversely related to forced expiratory volume in 1 s (FEV_1_) (Barcelo et al., 2006). Several studies have shown that IL-13 is expressed in bronchial biopsies in patients with asthma (Kroegel et al., 1996; Naseer et al., 1997; Berry et al., 2004; Saha et al., 2008). IL-13^+^ ILC2 were increased in the circulation of asthmatic patients with levels correlating with asthma severity (Jia et al., 2016). High production of IL-13 by cord blood CD4^+^ T cells is a predictor of development of atopic disorders (Martinez 2002).

### Polymorphisms in the Interleukin 13/Interleukin 4 Receptor Complex Associated With Asthma

Polymorphisms have been identified both in the IL-13 promoter (IL-13-1112 T) and the coding region (IL-13+Arg 130Gen, IL-13+2044G > A) in asthmatic patients (Hershey et al., 1997; Graves et al., 2000; Heinzmann et al., 2000; Arima et al., 2002; Cameron et al., 2006). One of the polymorphisms (Arg 130 Gln) (A/G) identified occur in the region critical for receptor-ligand interactions. Multiple polymorphism in IL-4R gene have also been identified and associated with asthma (Hershey et al., 1997; Kruse et al., 1999; Ober et al., 2000). Recently, three meta-analyses of IL-13 polymorphisms in adults and children suggested that the IL-13+1923 C/T polymorphism is associated with increased risk of asthma (Liu et al., 2014; Mei and Qu, 2017; Xu et al., 2017).

### Tralokinumab and Lebrikizumab for the Treatment of Severe Uncontrolled Asthma

Several anti-IL-13 mAbs (anrukinzumab, lebrikizumab, tralokinumab) have provided an opportunity to investigate the role of this cytokine in the pathophysiology of severe asthma, as well as assessing treatment response. A phase 1 study evaluated the pharmacokinetics, safety, and tolerability of tralokinumab in asthmatic patients receiving three different i.v. doses: 1 mg/kg, 5 mg/kg, or 10 mg/kg (Singh et al., 2010). Despite the small sample size, pharmacokinetics were linear over the dose range studied. The half-life was found to be 2–3 weeks and tralokinumab exhibited an acceptable safety profile.

A phase 2a, randomized, double-blind, placebo-controlled, parallel-group, multicenter study investigated the effects of different dose regimens of tralokinumab in 194 adults with moderate-to-severe asthma inadequately controlled with standard therapy (Piper et al., 2013). Three dose regimens were evaluated: 47 patients received s.c. tralokinumab 150 mg, 51 patients received tralokinumab 300 mg, 48 subjects received tralokinumab 600 mg, and 48 patients received placebo. The primary endpoint was the change from baseline in mean Asthma Control Questionnaire score (ACQ-6) at week 13. Secondary endpoints were change in FEV_1_, pre-bronchodilator lung function, patient-reported outcomes (PROs), rescue β_2_-agonist use, and safety outcomes. Mean ACQ-6 score improved in all treatment groups, from baseline to week 13. These changes in ACQ-6 persisted through week 24 and were greater in active patients with higher IL-13 sputum concentrations compared with subjects with lower IL-13 sputum concentrations or subjects receiving placebo. Improvement in FEV_1_ were higher in patients with peripheral eosinophil counts ≥ 300 cells/ml. Pulmonary function improvements were higher in tralokinumab patients with higher sputum IL-13 (≥ 10 pg/ml^-1^) compared to tralokinumab patients with lower sputum IL-13 (≤10 pg/ml^-1^) and patients receiving placebo. However, there was no significant difference in asthma exacerbation rate. The authors defined asthma exacerbations as either a progressive increase of asthma symptoms (cough, wheeze, chest tightness, and/or shortness of breath) or a reduction of >20% in peak expiratory flow or FEV_1_ from baseline that did not resolve after the initiation of rescue medications and resulted in an administration of systemic glucocorticoids.

The phase 2b clinical trial run by Brightling and colleagues evaluated safety and tolerability profile and the reduction in exacerbation rate and FEV_1_ improvement (Brightling et al., 2015). The authors randomized 452 severe asthmatic patients, all with two-to-six asthma exacerbations in the previous year, to receive tralokinumab (300 mg s.c. either every 2 weeks or every 2 weeks for 3 months and then every 4 weeks) or placebo as add-on therapy for 1 year. At the end of the study, they reported no changes in the annual exacerbation rate at week 52 (primary endpoint) in patients treated with tralokinumab, either every 2 or 4 weeks, *versus* placebo or in time to first exacerbation. The authors defined asthma exacerbation as an increase in asthma symptoms resulting in use or increase in dose of systemic glucocorticoids for three or more consecutive days. Similarly, secondary endpoints such as improvements in prebronchodilator FEV_1_, ACQ-6, and AQLQ(S) were not significant in patients treated with tralokinumab compared with placebo. They found a significant improvement in FEV_1_ in patients treated with tralokinumab every 2 weeks. In this study measurement of serum dipeptidyl peptidase-4 (DPP-4) and periostin concentrations were included as a predictive candidate biomarkers before the study was unmasked. Subgroup analyses of patients receiving tralokinumab every 2 weeks with airway reversibility at baseline, but not receiving oral glucocorticoids, showed some clinical improvements in the subgroup who had raised serum DPP-4 and periostin (Brightling et al., 2015). These preliminary results suggested that certain subpopulations of patients with severe asthma might respond to tralokinumab treatment.

A phase 2 multicenter, double-blind, randomized, placebo-controlled trial evaluated the effects of tralokinumab on eosinophilic airway inflammation in uncontrolled moderate-to-severe asthma (MESOS) (Russell et al., 2018). In this study, participants aged 18–75 years were randomly assigned to receive tralokinumab (300 mg s.c. every 2 weeks) or placebo. The primary outcome measure was change from baseline to week 12 in bronchial biopsy eosinophil count. Secondary outcome measures included change in blood and sputum eosinophil counts. Exploratory outcomes included FeNO and blood IgE concentrations. Tralokinumab did not affect bronchial, peripheral blood, or eosinophil counts compared to placebo at week 12. FeNO concentrations and total blood IgE were significantly reduced. The authors concluded that IL-13 is not crucial for eosinophilic airway inflammation control in patients with moderate-to-severe asthma.

Two large phase 3 clinical trials, STRATOS 1 and STRATOS 2, explored the use of periostin and DPP-4 as biomarkers of IL-13-driven inflammatory patterns in patients aged 12–75 years with severe uncontrolled asthma treated with tralokinumab (300 mg s.c. every 2 weeks for 52 weeks) or placebo (Panettieri et al., 2018). The primary endpoint was the annualized asthma exacerbation rate reduction at week 52 in the all-comers population for STRATOS 1 and in the biomarker-positive population for STRATOS 2. The results of both trials confirmed that tralokinumab did not improve the annual asthma exacerbation rate in the all-comers population with severe asthma. In contrast to the preliminary results of the phase 2 trial (Brightling et al., 2015), periostin and DPP-4 were not shown to predict response to tralokinumab. In both trials, tralokinumab-treated participants had a small increase in blood eosinophil counts from baseline, whereas placebo-treated participants did not. A recent meta-analysis of six randomized clinical trials suggested that tralokinumab was well tolerated and modestly improved FEV_1_ but did not reduce asthma exacerbations in severe uncontrolled asthma (Zhang et al., 2019).

Given the need to reduce oral glucocorticoid administration in patients with severe asthma, treatments that may allow tapering of glucocorticoids without loss of disease control are needed. A 40-week, randomized, double-blind trial (TROPOS) evaluated the oral glucocorticoid-sparing potential of tralokinumab in patients with severe, uncontrolled asthma requiring maintenance glucocorticoid treatment plus ICS/LABA (Busse et al., 2019). One hundred forty patients were randomized to tralokinumab (300 mg s.c. every 2 weeks) or placebo. The primary endpoint was percentage change from baseline in average glucocorticoid dose at week 40, while maintaining asthma control. Secondary endpoints included patients with a prescribed maintenance glucocorticoid dose of ≤ 5 mg, those with greater than 50% reduction in prescribed maintenance glucocorticoids dose and annual asthma exacerbation rate. An asthma exacerbation was defined as worsening of asthma that required a temporary increase in systemic glucocorticoids for ⩾3 days or that resulted in an emergency-room or urgent-care visit that led to a temporary increase in systemic glucocorticoids for ⩾3 days to treat symptoms or an inpatient hospitalization due to asthma. There were no significant between-group differences for primary and secondary endpoints. Reporting of adverse events and serious adverse events were similar for the tralokinumab and placebo groups.

A randomized, double-blind, placebo controlled study examined the effects of another anti-IL-13 mAb, lebrikizumab (250 mg s.c. once monthly for 6 months), on change in prebronchodilator FEV_1_ from baseline to week 12 in 219 adults with uncontrolled asthma (Corren et al., 2011). Lebrikizumab treatment was associated with greater improvement in percent change in FEV_1_ in patients with high pretreatment levels of serum periostin compared to patients with low periostin levels. In two replicate studies (LUTE and VERSE) in patients with moderate-to-severe uncontrolled asthma, lebrikizumab (37.5, 125 or 250 mg s.c. every 4 weeks) reduced asthma exacerbation rate by 60% compared to placebo in periostin-high patients and by 5% in periostin-low patients (Hanania et al., 2015). In these studies the authors defined asthma exacerbation as new or increased asthma symptoms that led to treatment with systemic glucocorticoids or to hospitalization. Two replicate, phase 3 trials (LAVOLTA1 and LAVOLTA2) explored the use of periostin and eosinophilia (≥ 300 cells/µl) as biomarkers of IL-13-driven inflammatory patterns in patients with severe uncontrolled asthma treated with lebrikizumab (37.5 mg or 125 mg s.c. once every 4 weeks for 52 weeks or placebo) (Hanania et al., 2016). The primary endpoint was the reduction in the rate of asthma exacerbations over 52 weeks in biomarker-high patients (periostin ≥ 50 ng/ml or blood eosinophils ≥ 300 cells/µl. In contrast to the preliminary results of phase 2 trials (Corren et al., 2011; Hanania et al., 2015), lebrikizumab did not consistently show significant reduction in asthma exacerbations in biomarker-high patients.

### Anti-Interleukin 13 in Nasal Polyposis

Chronic rhinosinusitis with nasal polyps (CRSwNP) is a common and relevant comorbidity for severe asthma (Heffler et al., 2013). The prevalence of CRSwNP is greater in asthmatics compared to the general population (Settipane and Chafee, 1977) and it increases with the severity of asthma (Pearlman et al., 2009; Lin, 2011), with the highest prevalence rates in non-atopic, late-onset, severe asthmatics (Amelink et al., 2013). More than 60% of patients with CRSwNP have asthma (Ragab et al., 2004; Guida et al., 2010). Moreover, CRWwNP is one of the significant determinants of poor asthma control (Heffler et al., 2013) and a hallmark of refractory eosinophilic asthma (Amelink et al., 2013). The clinical evidence of a strong relationship between CRSwNP and severe asthma raises the possibility of a shared pathogenesis. In particular both diseases seem to be dependent on eosinophilic inflammation mediated by epithelial cytokines such as thymic stromal lymphopoietin (TSLP), IL-25, and IL-33 secreted as a consequence of epithelial damage (Boita et al., 2016; Metha 2016; Varricchi et al., 2018c). The latter activation is mediated by ILC2 which produce several type 2 cytokines such as IL-13 and IL-5 (Aron and Akbari 2017; Poposki et al., 2017). Recently, it has been reported that there were significant increases in IL-13, IL-13Rα1, and IL-13Rα2 mRNA and protein concentrations in nasal polyp epithelium (Liu et al., 2018). Moreover, IL-13 treatment resulted in mucus overproduction and impairment of ciliary function of human nasal epithelial cells. Therefore, an anti-IL-13 strategy, such as tralokinumab, may be helpful in the management of patients with nasal polyps. Unfortunately, there are no clinical trials of treatment of CRSwNP with anti-IL13 mAbs. Interestingly, patients treated with dupilumab, a mAb against IL-4Rα, the common receptor for both IL-4 and IL-13 had a significant reduction in polyp size and improvement in symptoms and nasal and olfactory function (Bachert et al., 2016).

## Concluding Remarks and Perspectives

Three phase 2 clinical trials (Piper et al., 2013; Brightling et al., 2015; Russell et al., 2018) and three phase 3 clinical trials (Panettieri et al., 2018; Busse et al., 2019) have shown that tralokinumab did not lower the annual exacerbation rate and did not improve ACQ-6 scores compared to placebo in severe uncontrolled asthmatic patients. These negative findings parallel the results of two replicate, phase 3 trials with lebrikizumab (Hanania et al., 2016). These negative results are surprising given the wide spectrum of pro-inflammatory and pro-fibrogenic activities of IL-13 in experimental models of asthma (Chu et al., 1998; Laporte et al., 2001; Komai et al., 2003; Kanoh et al., 2011) and in asthmatic patients (Huang et al., 1995; Kroegel et al., 1996; Boniface et al., 2003; Truyen et al., 2006; Saha et al., 2008; Jia et al., 2016).

There are several possible explanations of these negative findings. First, it is likely that IL-13 is not the main cytokine involved in the complex network of severe asthma pathogenesis, meaning blocking it alone is ineffective. Second, as shown in several trials (Brightling et al., 2015; Hanania et al., 2016; Panettieri et al., 2018), the biomarkers (e.g., periostin, DPP-4, peripheral eosinophil count) used to identify responders to anti-IL-13 therapy are not optimal. Third, in both experimental models (Walter et al., 2001) and clinical studies (Brightling et al., 2015; Hanania et al., 2016; Panettieri et al., 2018) blocking IL-13 appears to have no effect on reducing tissue or blood eosinophilia, the pathophysiological feature most closely linked to asthma exacerbations. Fourth, perhaps the route of administration (i.e., s.c.) and the size of the anti-IL-13 mAb particles are not ideal. For instance, it has been reported the preliminary efficacy of a nebulized inhaled anti-IL-13 mAb antigen-binding fragment in macaque model of asthma (Lightwood et al., 2018). Finally, we cannot exclude the possibility that some of these negative findings could be due to the inclusion in clinical trials of patients with Th2-low asthma.

Interestingly, the initial attempts to develop cytokine therapies for asthma focusing on antagonizing IL-4 were also unsuccessful (Hart et al., 2002; Steinke 2004), as was an attempt to block the combined receptor (Wenzel et al., 2007; Burmeister Getz et al., 2009). By contrast, dupilumab, which binds to IL-4Rα and consequently blocks both IL-4 and IL-13 signaling, decreases asthma exacerbations and improves respiratory symptoms in patients with persistent asthma (Castro et al., 2018; Rabe et al., 2018). These observations suggest that only the effective simultaneous blockade of signaling from two main cytokines (i.e., IL-4 and IL-13) is effective in the treatment of severe asthma. Using allergic preclinical models, it has been demonstrated that the combined blockade of the IL-13 and IL-33 pathways leads to a greater inhibition of type 2 inflammation over inhibition of either pathway alone (Ramirez-Carrozzi et al., 2017). Similarly, co-blockade of IL-13 and IL-25 attenuated AHR, eosinophil infiltration in the lung, and mucus hyperproduction in a mouse model of OVA-induced asthma (Zhang et al., 2017). Recently, a novel dual antagonist anti-TSLP/IL-13 bispecific antibody has been described (Venkataramani et al., 2018). It will be interesting to see whether combinatorial blockade of multiple cytokines, including IL-13, may yield additional efficacy over single-axis therapies alone.

It is intriguing that IL-13 blockade modulates several aspects of different experimental models of allergic asthma (Laporte et al., 2001; Walter et al., 2001; Komai et al., 2003; Munitz et al., 2008; Chen et al., 2013; Ma et al., 2013; Chachi et al., 2017). Moreover, recent results demonstrate that anti-IL-13 antibody improves bronchial hyperresponsiveness and mucus production in a mouse model of non-allergic asthma (Gungl et al., 2018). These positive experimental results contrast with negative results in the treatment of asthmatic patients with different anti-IL-13 mAbs (tralokinumab and lebrikizumab) (Piper et al., 2013; Brightling et al., 2015; Hanania et al., 2016; Panettieri et al., 2018; Russell et al., 2018; Busse et al., 2019). These findings highlight that the results from murine studies do not always predict clinical effectiveness.

In conclusion, despite several efforts, attempts to demonstrate a benefit of anti-IL-13 in patients with severe asthma remain unproven. While this may be because the right predictive biomarkers or patient phenotypes have not yet been identified, it is perhaps more likely that there is enough redundancy in the pathophysiology of severe asthma to persist without IL-13. This is not to say, at this stage, that another clinical indication might not be found for IL-13-blocking drugs in future.

## Author Contributions

All authors contributed to reviewing the current literature and writing of the manuscript and approved the final version of the paper. Conceptualization: GM, GS, GV. Original draft preparation: GM, GS, GV. Final editing: GM, FG, VP, AP, EH, SL, GS, GV.

## Funding

This work was supported in part by grants from CISI-Lab Project (University of Naples Federico II) and TIMING Project (Regione Campania).

## Conflict of Interest

EH is advisory board member for AstraZeneca, Sanofi- Genzyme, Novartis, GSK, Circassia, Nestlè, and Purina.

The remaining authors declare that the research was conducted in the absence of any commercial or financial relationships that could be construed as a potential conflict of interest.

## Abbreviations

ACQ, asthma control questionnaire; AHR, airway hypersensitivity; BAL, bronchoalveolar lavage; CRSwNP, chronic sinusitis with nasal polyps; DC, dendritic cell; ERK, extracellular signal-regulated kinase; DPP-4, dipeptidyl peptidase-4; FcεRI, high affinity receptor for IgE; FeNO, fraction of exhaled nitric oxide; FEV1, forced expiratory volume in the first second; ILC, innate lymphoid cell; JAK, Janus kinase; LABA, long-acting β2-agonist; ICS, inhaled glucocorticoid; mAb, monoclonal antibody; OVA, ovalbumin; PEF, peak expiratory flow; PGD2, prostaglandin D2; PRO, patient-reported outcomes; SABA, short-acting β2-agonist; STAT6, signal transducer and activator of transcription; TSLP, thymic stromal lymphopoietin; TYK2, tyrosine kinase 2; VCAM, vascular cell adhesion molecule.

## References

[B1] Akaiwa M. Yu B. Umeshita-Suyama R. Terada N. Suto H. Koga T. (2001). Localization of human interleukin 13 receptor in non-haematopoietic cells. Cytokine 13, 75–84. 10.1006/cyto.2000.0814 11145846

[B2] Alasandagutti M. L. Ansari M. S. Sagurthi S. R. Valluri V. Gaddam S. (2017). Role of IL-13 genetic variants in signalling of asthma. Inflammation 40, 566–577. 10.1007/s10753-016-0503-3 28083766

[B3] Amelink M. de Groot J. C. de Nijs S. B. Lutter R. Zwinderman A. H. Sterk P. J. (2013). Severe adult-onset asthma: a distinct phenotype. J. Allergy Clin. Immunol. 132, 336–341. 10.1016/j.jaci.2013.04.052 23806634

[B4] Arima K. Umeshita-Suyama R. Sakata Y. Akaiwa M. Mao X. Q. Enomoto T. (2002). Upregulation of IL-13 concentration in vivo by the IL13 variant associated with bronchial asthma. J. Allergy Clin. Immunol. 109, 980–987. 10.1067/mai.2002.124656 12063528

[B5] Aron J. L. Akbari O. (2017). Regulatory T cells and type 2 innate lymphoid cell-dependent asthma. Allergy 72, 1148–1155. 10.1111/all.13139 28160290

[B6] Bachert C. Mannent L. Naclerio R. M. Mullol J. Ferguson B. J. Gevaert P. (2016). Effect of subcutaneous dupilumab on nasal polyp burden in patients with chronic sinusitis and nasal polyposis: a randomized clinical trial. JAMA 315, 469–479. 10.1001/jama.2015.19330 26836729

[B7] Bagnasco D. Ferrando M. Varricchi G. Passalacqua G. Canonica G. W. (2016). A Critical Evaluation of Anti-IL-13 and Anti-IL-4 Strategies in Severe Asthma. Int. Arch. Allergy Immunol. 170, 122–131. 10.1159/000447692 27637004

[B8] Bagnasco D. Ferrando M. Varricchi G. Puggioni F. Passalacqua G. Canonica G. W. (2017). Anti-Interleukin 5 (IL-5) and IL-5Ra biological drugs: efficacy, safety, and future perspectives in severe eosinophilic asthma. Front. Med. (Lausanne) 4, 135. 10.3389/fmed.2017.00135 28913336 PMC5583162

[B9] Barcelo B. Pons J. Fuster A. Sauleda J. Noguera A. Ferrer J. M. (2006). Intracellular cytokine profile of T lymphocytes in patients with chronic obstructive pulmonary disease. Clin. Exp. Immunol. 145, 474–479. 10.1111/j.1365-2249.2006.03167.x 16907916 PMC1809717

[B10] Bel E. H. Wenzel S. E. Thompson P. J. Prazma C. M. Keene O. N. Yancey S. W. (2014). Oral glucocorticoid-sparing effect of mepolizumab in eosinophilic asthma. N. Engl. J. Med. 371, 1189–1197. 10.1056/NEJMoa1403291 25199060

[B11] Berry M. A. Parker D. Neale N. Woodman L. Morgan A. Monk P. (2004). Sputum and bronchial submucosal IL-13 expression in asthma and eosinophilic bronchitis. J. Allergy Clin. Immunol. 114, 1106–1109. 10.1016/j.jaci.2004.08.032 15536417

[B12] Bhattacharjee A. Shukla M. Yakubenko V. P. Mulya A. Kundu S. Cathcart M. K. (2013). IL-4 and IL-13 employ discrete signaling pathways for target gene expression in alternatively activated monocytes/macrophages. Free Radic. Biol. Med. 54, 1–16. 10.1016/j.freeradbiomed.2012.10.553 23124025 PMC3534796

[B13] Bjermer L. Lemiere C. Maspero J. Weiss S. Zangrilli J. Germinaro M. (2016). Reslizumab for inadequately controlled asthma with elevated blood eosinophil levels: a randomized phase 3 study. Chest 150, 789–798. 10.1016/j.chest.2016.03.032 27056586

[B14] Bochner B. S. Klunk D. A. Sterbinsky S. A. Coffman R. L. Schleimer R. P. (1995). IL-13 selectively induces vascular cell adhesion molecule-1 expression in human endothelial cells. J. Immunol. 154, 799–803. 7529288

[B15] Bodey K. J. Semper A. E. Redington A. E. Madden J. Teran L. M. Holgate S. T. (1999). Cytokine profiles of BAL T cells and T-cell clones obtained from human asthmatic airways after local allergen challenge. Allergy 54, 1083–1093. 10.1034/j.1398-9995.1999.00889.x 10536887

[B16] Boita M. Bucca C. Riva G. Heffler E. Rolla G. (2016). Release of Type 2 Cytokines by Epithelial Cells of Nasal Polyps. J. Immunol. Res. 2016, 2643297. 10.1155/2016/2643297 28127565 PMC5227162

[B17] Boniface S. Koscher V. Mamessier E. El Biaze M. Dupuy P. Lorec A. M. (2003). Assessment of T lymphocyte cytokine production in induced sputum from asthmatics: a flow cytometry study. Clin. Exp. Allergy 33, 1238–1243. 10.1046/j.1365-2222.2003.01762.x 12956745

[B18] Borriello F. Longo M. Spinelli R. Pecoraro A. Granata F. Staiano R. I. (2015). IL-3 synergises with basophil-derived IL-4 and IL-13 to promote the alternative activation of human monocytes. Eur. J. Immunol. 45, 2042–2051. 10.1002/eji.201445303 25824485 PMC4496336

[B19] Borriello F. Galdiero M. R. Varricchi G. Loffredo S. Spadaro G. Marone G. (2019). Innate Immune Modulation by GM-CSF and IL-3 in Health and Disease. Int. J. Mol. Sci. 20, E834. 10.3390/ijms20040834 30769926 PMC6412223

[B20] Brightling C. E. Chanez P. Leigh R. O'Byrne P. M. Korn S. She D. (2015). Efficacy and safety of tralokinumab in patients with severe uncontrolled asthma: a randomised, double-blind, placebo-controlled, phase 2b trial. Lancet Respir. Med. 3, 692–701. 10.1016/S2213-2600(15)00197-6 26231288

[B21] Burd P. R. Thompson W. C. Max E. E. Mills F. C. (1995). Activated mast cells produce interleukin 13. J. Exp. Med. 181, 1373–1380. 10.1084/jem.181.4.1373 7535336 PMC2191950

[B22] Burmeister Getz E. Fisher D. M. Fuller R. (2009). Human pharmacokinetics/pharmacodynamics of an interleukin-4 and interleukin-13 dual antagonist in asthma. J. Clin. Pharmacol. 49, 1025–1036. 10.1177/0091270009341183 19717725

[B23] Busse W. W. Brusselle G. G. Korn S. Kuna P. Magnan A. Cohen D. (2019). Tralokinumab did not demonstrate oral corticosteroid-sparing effects in severe asthma. Eur. Respir. J. 53, 1800948. 10.1183/13993003.00948-2018 30442714

[B24] Cameron L. Webster R. B. Strempel J. M. Kiesler P. Kabesch M. Ramachandran H. (2006). Th2 cell-selective enhancement of human IL13 transcription by IL13-1112C>T, a polymorphism associated with allergic inflammation. J. Immunol. 177, 8633–8642. 10.4049/jimmunol.177.12.8633 17142763 PMC11507172

[B25] Cao H. Zhang J. Liu H. Wan L. Zhang H. Huang Q. (2016). IL-13/STAT6 signaling plays a critical role in the epithelial-mesenchymal transition of colorectal cancer cells. Oncotarget 7, 61183–61198. 10.18632/oncotarget.11282 27533463 PMC5308644

[B26] Castro M. Corren J. Pavord I. D. Maspero J. Wenzel S. Rabe K. F. (2018). Dupilumab efficacy and safety in moderate-to-severe uncontrolled asthma. N. Engl. J. Med. 378, 2486–2496. 10.1056/NEJMoa1804092 29782217

[B27] Chachi L. Shikotra A. Duffy S. M. Tliba O. Brightling C. Bradding P. (2013). Functional KCa3.1 channels regulate steroid insensitivity in bronchial smooth muscle cells. J. Immunol. 191, 2624–2636. 10.4049/jimmunol.1300104 23904164 PMC3753579

[B28] Chachi L. Abbasian M. Gavrila A. Alzahrani A. Tliba O. Bradding P. (2017). Protein phosphatase 5 mediates corticosteroid insensitivity in airway smooth muscle in patients with severe asthma. Allergy 72, 126–136. 10.1111/all.13003 27501780 PMC5154839

[B29] Chatila T. A. (2004). Interleukin-4 receptor signaling pathways in asthma pathogenesis. Trends Mol. Med. 10, 493–499. 10.1016/j.molmed.2004.08.004 15464449

[B30] Chen W. Sivaprasad U. Tabata Y. Gibson A. M. Stier M. T. Finkelman F. D. (2009). IL-13R alpha 2 membrane and soluble isoforms differ in humans and mice. J. Immunol. 183, 7870–7876. 10.4049/jimmunol.0901028 20007572 PMC2822278

[B31] Chen W. Sivaprasad U. Gibson A. M. Ericksen M. B. Cunningham C. M. Bass S. A. (2013). IL-13 receptor alpha2 contributes to development of experimental allergic asthma. J. Allergy Clin. Immunol. 132, 951–958 e951-956. 10.1016/j.jaci.2013.04.016 23763980 PMC3836839

[B32] Choy D. F. Hart K. M. Borthwick L. A. Shikotra A. Nagarkar D. R. Siddiqui S. (2015). TH2 and TH17 inflammatory pathways are reciprocally regulated in asthma. Sci. Transl. Med. 7, 301ra129. 10.1126/scitranslmed.aab3142 26290411

[B33] Chu H. W. Halliday J. L. Martin R. J. Leung D. Y. Szefler S. J. Wenzel S. E. (1998). Collagen deposition in large airways may not differentiate severe asthma from milder forms of the disease. Am. J. Respir. Crit. Care Med. 158, 1936–1944. 10.1164/ajrccm.158.6.9712073 9847289

[B34] Corne J. Chupp G. Lee C. G. Homer R. J. Zhu Z. Chen Q. (2000). IL-13 stimulates vascular endothelial cell growth factor and protects against hyperoxic acute lung injury. J. Clin. Invest. 106, 783–791. 10.1172/JCI9674 10995789 PMC381393

[B35] Corren J. Lemanske R. F. Hanania N. A. Korenblat P. E. Parsey M. V. Arron J. R. (2011). Lebrikizumab treatment in adults with asthma. N. Engl. J. Med. 365, 1088–1098. 10.1056/NEJMoa1106469 21812663

[B36] Cosmi L. Annunziato F. Galli M. I. G. Maggi R. M. E. Nagata K. Romagnani S. (2000). CRTH2 is the most reliable marker for the detection of circulating human type 2 Th and type 2 T cytotoxic cells in health and disease. Eur. J. Immunol. 30, 2972–2979. 10.1002/1521-4141(200010)30:10<2972::AID-IMMU2972>3.0.CO;2- 11069080

[B37] Dakhama A. Collins M. L. Ohnishi H. Goleva E. Leung D. Y. Alam R. (2013). IL-13-producing BLT1-positive CD8 cells are increased in asthma and are associated with airway obstruction. Allergy 68, 666–673. 10.1111/all.12135 23573812 PMC3630251

[B38] de Vries J. E. (1998). The role of IL-13 and its receptor in allergy and inflammatory responses. J. Allergy Clin. Immunol. 102, 165–169. 10.1016/S0091-6749(98)70080-6 9723655

[B39] Detoraki A. Staiano R. I. Granata F. Giannattasio G. Prevete N. de Paulis A. (2009). Vascular endothelial growth factors synthesized by human lung mast cells exert angiogenic effects. J. Allergy Clin. Immunol. 123, 1142–1149, 1149 e1141-1145. 10.1016/j.jaci.2009.01.044 19275959

[B40] Detoraki A. Granata F. Staibano S. Rossi F. W. Marone G. Genovese A. (2010). Angiogenesis and lymphangiogenesis in bronchial asthma. Allergy 65, 946–958. 10.1111/j.1398-9995.2010.02372.x 20415716

[B41] Doucet C. Brouty-Boye D. Pottin-Clemenceau C. Canonica G. W. Jasmin C. Azzarone B. (1998). Interleukin (IL) 4 and IL-13 act on human lung fibroblasts. Implication in asthma. J. Clin. Invest. 101, 2129–2139. 10.1172/JCI741 9593769 PMC508801

[B42] Fahy J. V. (2015). Type 2 inflammation in asthma–present in most, absent in many. Nat. Rev. Immunol. 15, 57–65. 10.1038/nri3786 25534623 PMC4390063

[B43] Fichtner-Feigl S. Strober W. Kawakami K. Puri R. K. Kitani A. (2006). IL-13 signaling through the IL-13alpha2 receptor is involved in induction of TGF-beta1 production and fibrosis. Nat. Med. 12, 99–106. 10.1038/nm1332 16327802

[B44] Finkelman F. D. Shea-Donohue T. Morris S. C. Gildea L. Strait R. Madden K. B. (2004). Interleukin-4- and interleukin-13-mediated host protection against intestinal nematode parasites. Immunol. Rev. 201, 139–155. 10.1111/j.0105-2896.2004.00192.x 15361238

[B45] FitzGerald J. M. Bleecker E. R. Nair P. Korn S. Ohta K. Lommatzsch M. (2016). Benralizumab, an anti-interleukin-5 receptor alpha monoclonal antibody, as add-on treatment for patients with severe, uncontrolled, eosinophilic asthma (CALIMA): a randomised, double-blind, placebo-controlled phase 3 trial. Lancet 388, 2128–2141. 10.1016/S0140-6736(16)31322-8 27609406

[B46] FitzGerald J. M. Bleecker E. R. Menzies-Gow A. Zangrilli J. G. Hirsch I. Metcalfe P. (2018). Predictors of enhanced response with benralizumab for patients with severe asthma: pooled analysis of the SIROCCO and CALIMA studies. Lancet Respir. Med. 6, 51–64. 10.1016/S2213-2600(17)30344-2 28919200

[B47] Fujisawa T. Joshi B. Nakajima A. Puri R. K. (2009). A novel role of interleukin-13 receptor alpha2 in pancreatic cancer invasion and metastasis. Cancer Res. 69, 8678–8685. 10.1158/0008-5472.CAN-09-2100 19887609

[B48] Fushimi T. Okayama H. Shimura S. Saitoh H. Shirato K. (1998). Dexamethasone suppresses gene expression and production of IL-13 by human mast cell line and lung mast cells. J. Allergy Clin. Immunol. 102, 134–142. 10.1016/S0091-6749(98)70064-8 9679857

[B49] Gandhi N. A. Pirozzi G. Graham N. M. H. (2017). Commonality of the IL-4/IL-13 pathway in atopic diseases. Expert Rev. Clin. Immunol. 13, 425–437. 10.1080/1744666X.2017.1298443 28277826

[B50] Gauvreau G. M. Boulet L. P. Cockcroft D. W. Fitzgerald J. M. Carlsten C. Davis B. E. (2011). Effects of interleukin-13 blockade on allergen-induced airway responses in mild atopic asthma. Am. J. Respir. Crit. Care Med. 183, 1007–1014. 10.1164/rccm.201008-1210OC 21057005

[B51] Genovese A. Borgia G. Bjorck L. Petraroli A. de Paulis A. Piazza M. (2003). Immunoglobulin superantigen protein L induces IL-4 and IL-13 secretion from human Fc epsilon RI+ cells through interaction with the kappa light chains of IgE. J. Immunol. 170, 1854–1861. 10.4049/jimmunol.170.4.1854 12574351

[B52] Gibbs B. F. Haas H. Falcone F. H. Albrecht C. Vollrath I. B. Noll T. (1996). Purified human peripheral blood basophils release interleukin-13 and preformed interleukin-4 following immunological activation. Eur. J. Immunol. 26, 2493–2498. 10.1002/eji.1830261033 8898965

[B53] Gould H. J. Sutton B. J. (2008). IgE in allergy and asthma today. Nat. Rev. Immunol. 8, 205–217. 10.1038/nri2273 18301424

[B54] Gour N. Wills-Karp M. (2015). IL-4 and IL-13 signaling in allergic airway disease. Cytokine 75, 68–78. 10.1016/j.cyto.2015.05.014 26070934 PMC4532591

[B55] Graves P. E. Kabesch M. Halonen M. Holberg C. J. Baldini M. Fritzsch C. (2000). A cluster of seven tightly linked polymorphisms in the IL-13 gene is associated with total serum IgE levels in three populations of white children. J. Allergy Clin. Immunol. 105, 506–513. 10.1067/mai.2000.104940 10719301

[B56] Grunig G. Warnock M. Wakil A. E. Venkayya R. Brombacher F. Rennick D. M. (1998). Requirement for IL-13 independently of IL-4 in experimental asthma. Science 282, 2261–2263. 10.1126/science.282.5397.2261 9856950 PMC3897229

[B57] Grunstein M. M. Hakonarson H. Leiter J. Chen M. Whelan R. Grunstein J. S. (2002). IL-13-dependent autocrine signaling mediates altered responsiveness of IgE-sensitized airway smooth muscle. Am. J. Physiol. Lung Cell Mol. Physiol. 282, L520–L528. 10.1152/ajplung.00343.2001 11839548

[B58] Guida G. Rolla G. Badiu I. Marsico P. Pizzimenti S. Bommarito L. (2010). Determinants of exhaled nitric oxide in chronic rhinosinusitis. Chest 137, 658–664. 10.1378/chest.09-0667 19837820

[B59] Gungl A. Biasin V. Wilhelm J. Olschewski A. Kwapiszewska G. Marsh L. M. (2018). Fra2 Overexpression in Mice Leads to Non-allergic Asthma Development in an IL-13 Dependent Manner. Front. Immunol. 9, 2018. 10.3389/fimmu.2018.02018 30233597 PMC6133984

[B60] Hajoui O. Janani R. Tulic M. Joubert P. Ronis T. Hamid Q. (2004). Synthesis of IL-13 by human B lymphocytes: regulation and role in IgE production. J. Allergy Clin. Immunol. 114, 657–663. 10.1016/j.jaci.2004.05.034 15356573

[B61] Hanania N. A. Noonan M. Corren J. Korenblat P. Zheng Y. Fischer S. K. (2015). Lebrikizumab in moderate-to-severe asthma: pooled data from two randomised placebo-controlled studies. Thorax 70, 748–756. 10.1136/thoraxjnl-2014-206719 26001563 PMC4515999

[B62] Hanania N. A. Korenblat P. Chapman K. R. Bateman E. D. Kopecky P. Paggiaro P. (2016). Efficacy and safety of lebrikizumab in patients with uncontrolled asthma (LAVOLTA I and LAVOLTA II): replicate, phase 3, randomised, double-blind, placebo-controlled trials. Lancet Respir. Med. 4, 781–796. 10.1016/S2213-2600(16)30265-X 27616196

[B63] Hancock A. Armstrong L. Gama R. Millar A. (1998). Production of interleukin 13 by alveolar macrophages from normal and fibrotic lung. Am. J. Respir. Cell Mol. Biol. 18, 60–65. 10.1165/ajrcmb.18.1.2627 9448046

[B64] Hart T. K. Blackburn M. N. Brigham-Burke M. Dede K. Al-Mahdi N. Zia-Amirhosseini P. (2002). Preclinical efficacy and safety of pascolizumab (SB 240683): a humanized anti-interleukin-4 antibody with therapeutic potential in asthma. Clin. Exp. Immunol. 130, 93–100. 10.1046/j.1365-2249.2002.01973.x 12296858 PMC1906490

[B65] He C. H. Lee C. G. Dela Cruz C. S. Lee C. M. Zhou Y. Ahangari F. (2013). Chitinase 3-like 1 regulates cellular and tissue responses *via* IL-13 receptor alpha2. Cell Rep. 4, 830–841. 10.1016/j.celrep.2013.07.032 23972995 PMC3988532

[B66] Heffler E. Pizzimenti S. Badiu I. Guida G. Ricciardolo F. L. Bucca C. (2013). Nasal nitric oxide is a marker of poor asthma control. J. Breath Res. 7, 026009. 10.1088/1752-7155/7/2/026009 23665726

[B67] Heinzmann A. Mao X. Q. Akaiwa M. Kreomer R. T. Gao P. S. Ohshima K. (2000). Genetic variants of IL-13 signalling and human asthma and atopy. Hum. Mol. Genet. 9, 549–559. 10.1093/hmg/9.4.549 10699178

[B68] Hershey G. K. Friedrich M. F. Esswein L. A. Thomas M. L. Chatila T. A. (1997). The association of atopy with a gain-of-function mutation in the alpha subunit of the interleukin-4 receptor. N. Engl. J. Med. 337, 1720–1725. 10.1056/NEJM199712113372403 9392697

[B69] Hershey G. K. (2003). IL-13 receptors and signaling pathways: an evolving web. J. Allergy Clin. Immunol. 111, 677–690. 10.1067/mai.2003.1333 12704343

[B70] Hew M. Gillman A. Sutherland M. Wark P. Bowden J. Guo M. (2016). Real-life effectiveness of omalizumab in severe allergic asthma above the recommended dosing range criteria. Clin. Exp. Allergy 46, 1407–1415. 10.1111/cea.12774 27377155

[B71] Hinks T. S. Brown T. Lau L. C. Rupani H. Barber C. Elliott S. (2016). Multidimensional endotyping in patients with severe asthma reveals inflammatory heterogeneity in matrix metalloproteinases and chitinase 3-like protein 1. J. Allergy Clin. Immunol. 138, 61–75. 10.1016/j.jaci.2015.11.020 26851968 PMC4929135

[B72] Holgate S. T. Wenzel S. Postma D. S. Weiss S. T. Renz H. Sly P. D. (2015). Asthma. Nat. Rev. Dis. Primers. 1, 15025. 10.1038/nrdp.2015.25 27189668 PMC7096989

[B73] Horie S. Okubo Y. Hossain M. Sato E. Nomura H. Koyama S. (1997). Interleukin-13 but not interleukin-4 prolongs eosinophil survival and induces eosinophil chemotaxis. Intern. Med. 36, 179–185. 10.2169/internalmedicine.36.179 9144009

[B74] Huang S. K. Xiao H. Q. Kleine-Tebbe J. Paciotti G. Marsh D. G. Lichtenstein L. M. (1995). IL-13 expression at the sites of allergen challenge in patients with asthma. J. Immunol. 155, 2688–2694. 7650396

[B75] Ingram J. L. Kraft M. (2012). IL-13 in asthma and allergic disease: asthma phenotypes and targeted therapies. J. Allergy Clin. Immunol. 130, 829–842. 10.1016/j.jaci.2012.06.034 22951057

[B76] Jia Y. Fang X. Zhu X. Bai C. Zhu L. Jin M. (2016). IL-13(+) Type 2 innate lymphoid cells correlate with asthma control status and treatment response. Am. J. Respir. Cell Mol. Biol. 55, 675–683. 10.1165/rcmb.2016-0099OC 27314535

[B77] Kanoh S. Tanabe T. Rubin B. K. (2011). IL-13-induced MUC5AC production and goblet cell differentiation is steroid resistant in human airway cells. Clin. Exp. Allergy 41, 1747–1756. 10.1111/j.1365-2222.2011.03852.x 22092504

[B78] Kasaian M. T. Raible D. Marquette K. Cook T. A. Zhou S. Tan X. Y. (2011). IL-13 antibodies influence IL-13 clearance in humans by modulating scavenger activity of IL-13Ralpha2. J. Immunol. 187, 561–569. 10.4049/jimmunol.1100467 21622864

[B79] Kaur R. Chupp G. (2019). Phenotypes and endotypes of adult asthma: Moving toward precision medicine. J. Allergy Clin. Immunol. 144, 1–12. 10.1016/j.jaci.2019.05.031 31277742

[B80] Kaur D. Hollins F. Woodman L. Yang W. Monk P. May R. (2006). Mast cells express IL-13R alpha 1: IL-13 promotes human lung mast cell proliferation and Fc epsilon RI expression. Allergy 61, 1047–1053. 10.1111/j.1398-9995.2006.01139.x 16918506

[B81] Kawakami K. Taguchi J. Murata T. Puri R. K. (2001). The interleukin-13 receptor alpha2 chain: an essential component for binding and internalization but not for interleukin-13-induced signal transduction through the STAT6 pathway. Blood 97, 2673–2679. 10.1182/blood.V97.9.2673 11313257

[B82] Kerkhof M. Tran T. N. Soriano J. B. Golam S. Gibson D. Hillyer E. V. (2018). Healthcare resource use and costs of severe, uncontrolled eosinophilic asthma in the UK general population. Thorax 73, 116–124. 10.1136/thoraxjnl-2017-210531 28918400 PMC5801646

[B83] Khatri S. Moore W. Gibson P. G. Leigh R. Bourdin A. Maspero J. (2019). Assessment of the long-term safety of mepolizumab and durability of clinical response in patients with severe eosinophilic asthma. J. Allergy Clin. Immunol. 143, 1742–1751 e1747. 10.1016/j.jaci.2018.09.033 30359681

[B84] Kirstein F. Nieuwenhuizen N. E. Jayakumar J. Horsnell W. G. C. Brombacher F. (2016). Role of IL-4 receptor alpha-positive CD4(+) T cells in chronic airway hyperresponsiveness. J. Allergy Clin. Immunol. 137, 1852–1862 e1859. 10.1016/j.jaci.2015.10.036 26688514

[B85] Komai M. Tanaka H. Masuda T. Nagao K. Ishizaki M. Sawada M. (2003). Role of Th2 responses in the development of allergen-induced airway remodelling in a murine model of allergic asthma. Br. J. Pharmacol. 138, 912–920. 10.1038/sj.bjp.0705105 12642393 PMC1573716

[B86] Kondo M. Tamaoki J. Takeyama K. Isono K. Kawatani K. Izumo T. (2006). Elimination of IL-13 reverses established goblet cell metaplasia into ciliated epithelia in airway epithelial cell culture. Allergol. Int. 55, 329–336. 10.2332/allergolint.55.329 17075276

[B87] Kroegel C. Julius P. Matthys H. Virchow J. C. Jr. Luttmann W. (1996). Endobronchial secretion of interleukin-13 following local allergen challenge in atopic asthma: relationship to interleukin-4 and eosinophil counts. Eur. Respir. J. 9, 899–904. 10.1183/09031936.96.09050899 8793449

[B88] Kruse S. Japha T. Tedner M. Sparholt S. H. Forster J. Kuehr J. (1999). The polymorphisms S503P and Q576R in the interleukin-4 receptor alpha gene are associated with atopy and influence the signal transduction. Immunology 96, 365–371. 10.1046/j.1365-2567.1999.00705.x 10233717 PMC2326760

[B89] Kumar R. K. Herbert C. Webb D. C. Li L. Foster P. S. (2004). Effects of anticytokine therapy in a mouse model of chronic asthma. Am. J. Respir. Crit. Care Med. 170, 1043–1048. 10.1164/rccm.200405-681OC 15306533

[B90] Kuperman D. A. Huang X. Koth L. L. Chang G. H. Dolganov G. M. Zhu Z. (2002). Direct effects of interleukin-13 on epithelial cells cause airway hyperreactivity and mucus overproduction in asthma. Nat. Med. 8, 885–889. 10.1038/nm734 12091879

[B91] Lang D. M. Polansky M. (1994). Patterns of asthma mortality in Philadelphia from 1969 to 1991 . N. Engl. J. Med. 331, 1542–1546. 10.1056/NEJM199412083312302 7969323

[B92] Lange P. Ulrik C. S. Vestbo J. (1996). Mortality in adults with self-reported asthma. Copenhagen City Heart Study Group. Lancet 347, 1285–1289. 10.1016/S0140-6736(96)90937-X 8622503

[B93] Laporte J. C. Moore P. E. Baraldo S. Jouvin M. H. Church T. L. Schwartzman I. N. (2001). Direct effects of interleukin-13 on signaling pathways for physiological responses in cultured human airway smooth muscle cells. Am. J. Respir. Crit. Care Med. 164, 141–148. 10.1164/ajrccm.164.1.2008060 11435252

[B94] LaPorte S. L. Juo Z. S. Vaclavikova J. Colf L. A. Qi X. Heller N. M. (2008). Molecular and structural basis of cytokine receptor pleiotropy in the interleukin-4/13 system. Cell 132, 259–272. 10.1016/j.cell.2007.12.030 18243101 PMC2265076

[B95] Leigh R. Ellis R. Wattie J. Donaldson D. D. Inman M. D. (2004). Is interleukin-13 critical in maintaining airway hyperresponsiveness in allergen-challenged mice? Am. J. Respir. Crit. Care Med. 170, 851–856. 10.1164/rccm.200311-1488OC 15242841

[B96] Li L. Xia Y. Nguyen A. Lai Y. H. Feng L. Mosmann T. R. (1999). Effects of Th2 cytokines on chemokine expression in the lung: IL-13 potently induces eotaxin expression by airway epithelial cells. J. Immunol. 162, 2477–2487. 10072486

[B97] Lightwood D. Tservistas M. Zehentleitner M. Sarkar K. Turner A. Bracher M. (2018). Efficacy of an inhaled IL-13 antibody fragment in a model of chronic asthma. Am. J. Respir. Crit. Care Med. 198, 610–619. 10.1164/rccm.201712-2382OC 29883204

[B98] Lin D. C. (2011). Association between severity of asthma and degree of chronic rhinosinusitis. Am. J. Rhinol. Allergy 205–208. 10.2500/ajra.2011.25.3613 21819754 PMC3390198

[B99] Liu Z. Li P. Wang J. Fan Q. Yan P. Zhang X. (2014). A meta-nalysis of IL-13 polymorphisms and pediatric asthma risk. Med. Sci. Monit. 20, 2617–2623. 10.12659/MSM.891017 25502839 PMC4271802

[B100] Liu J. Li Y. Y. Andiappan A. K. Yan Y. Tan K. S. Ong H. H. (2018). Role of IL-13Ralpha2 in modulating IL-13-induced MUC5AC and ciliary changes in healthy and CRSwNP mucosa. Allergy 73, 1673–1685. 10.1111/all.13424 29405354

[B101] Lozano R. Naghavi M. Foreman K. Lim S. Shibuya K. Aboyans V. (2012). Global and regional mortality from 235 causes of death for 20 age groups in 1990 and 2010: a systematic analysis for the Global Burden of Disease Study 2010. Lancet 380, 2095–2128. 10.1016/S0140-6736(12)61728-0 23245604 PMC10790329

[B102] Lupardus P. J. Birnbaum M. E. Garcia K. C. (2010). Molecular basis for hared cytokine recognition revealed in the structure of an unusually high ffinity complex between IL-13 and IL-13Ralpha2. Structure 18, 332–342. 10.1016/j.str.2010.01.003 20223216 PMC2850121

[B103] Luttmann W. Knoechel B. Foerster M. Matthys H. Virchow J. C. Jr. Kroegel C. (1996). Activation of human eosinophils by IL-13. Induction of CD69 urface antigen, its relationship to messenger RNA expression, and promotion of ellular viability. J. Immunol. 157, 1678–1683. 8759755

[B104] Ma Y. HayGlass K. T. Becker A. B. Fan Y. Yang X. Basu S. (2007). Novel recombinant interleukin-13 peptide-based vaccine reduces airway llergic inflammatory responses in mice. Am. J. Respir. Crit. Care Med. 176, 439–445. 10.1164/rccm.200610-1405OC 17556715

[B105] Ma Y. Halayko A. J. Basu S. Guan Q. Weiss C. R. Ma A. G. (2013). Sustained suppression of IL-13 by a vaccine attenuates airway inflammation and remodeling in mice. Am. J. Respir. Cell Mol. Biol. 48, 540–549. 10.1165/rcmb.2012-0060OC 23470628

[B106] Macchia D. Melioli G. Pravettoni V. Nucera E. Piantanida M. Aminati M. (2015). Guidelines for the use and interpretation of diagnostic ethods in adult food allergy. Clin. Mol. Allergy 13, 27. 10.1186/s12948-15-0033-9 26441488 PMC4593201

[B107] Marone G. Triggiani M. de Paulis A. (2005). Mast cells and basophils: friends as well as foes in bronchial asthma? Trends Immunol. 26, 25–31. 10.1016/j.it.2004.10.010 15629406

[B108] Marone G. Galdiero M. R. Pecoraro A. Pucino V. Criscuolo G. Triassi M. (2019). Prostaglandin D2 receptor antagonists in allergic disorders: safety, efficacy, and future perspectives. Expert Opin. Invest. Drugs 28, 73–84. 10.1080/13543784.2019.1555237 30513028

[B109] Martinez F. D. (2002). What have we learned from the Tucson Children's espiratory Study? Paediatr. Respir. Rev. 3, 193–197. 10.1016/S1526-542(02)00188-4 12376055

[B110] Martinez-Nunez R. T. Louafi F. Sanchez-Elsner T. (2011). The nterleukin 13 (IL-13) pathway in human macrophages is modulated by icroRNA-155 *via* direct targeting of interleukin 13 receptor alpha1 IL13Ralpha1). J. Biol. Chem. 286, 1786–1794. 10.1074/jbc.M110.169367 21097505 PMC3023473

[B111] McCormick S. M. Heller N. M. (2015). Commentary: IL-4 and IL-13 receptors and signaling. Cytokine 75, 38–50. 10.1016/j.cyto.2015.05.023 26187331 PMC4546937

[B112] McKenzie G. J. Fallon P. G. Emson C. L. Grencis R. K. cKenzie A. N. (1999). Simultaneous disruption of interleukin (IL)-4 and IL-13 efines individual roles in T helper cell type 2-mediated responses. J. Exp. Med. 89, 1565–1572. 10.1084/jem.189.10.1565 PMC2193635 10330435

[B113] Mei Q. Qu J. (2017). Interleukin-13 +2044 G/A and +1923C/T olymorphisms are associated with asthma susceptibility in Asians: a meta-nalysis. Med. (Baltimore) 96, e9203. 10.1097/MD.0000000000009203 PMC5758167 29390465

[B114] Metha A. K. (2016). Rhinovirus infection interferes with induction of tolerance to aeroantigens through OX40 ligand, thymic stromal lymphopoietin, and IL-33. J. Allergy Clin. Immunol. 137, 278–288. 10.1016/j.jaci.2015.05.007 26100084 PMC4684822

[B115] Minty A. Chalon P. Derocq J. M. Dumont X. Guillemot J. C. Kaghad M. (1993). Interleukin-13 is a new human lymphokine regulating inflammatory and immune responses. Nature 362, 248–250. 10.1038/362248a0 8096327

[B116] Munitz A. Brandt E. B. Mingler M. Finkelman F. D. Rothenberg M. E. (2008). Distinct roles for IL-13 and IL-4 via IL-13 receptor alpha1 and the type II L-4 receptor in asthma pathogenesis. Proc. Natl. Acad. Sci. U. S. A. 105, 7240–7245. 10.1073/pnas.0802465105 18480254 PMC2386078

[B117] Naseer T. Minshall E. M. Leung D. Y. Laberge S. Ernst P. Martin R. J. (1997). Expression of IL-12 and IL-13 mRNA in asthma and their modulation in esponse to steroid therapy. Am. J. Respir. Crit. Care Med. 155, 845–851. 10.1164/ajrccm.155.3.9117015 9117015

[B118] Nilsson G. Nilsson K. (1995). Effects of interleukin (IL)-13 on immediate-early response gene expression, phenotype and differentiation of human mast ells. Comparison with IL-4. Eur. J. Immunol. 25, 870–873. 10.1002/eji.1830250337 7705421

[B119] Ober C. Leavitt S. A. Tsalenko A. Howard T. D. Hoki D. M. Daniel R. (2000). Variation in the interleukin 4-receptor alpha gene confers susceptibility to asthma and atopy in ethnically diverse populations. Am. J. Hum. Genet. 66, 517–526. 10.1086/302781 10677312 PMC1288105

[B120] Ochensberger B. Daepp G. C. Rihs S. Dahinden C. A. (1996). Human lood basophils produce interleukin-13 in response to IgE-receptor-dependent and independent activation. Blood 88, 3028–3037. 10.1182/blood.V88.8.3028.bloodjournal8883028 8874201

[B121] Oetjen L. K. Mack M. R. Feng J. Whelan T. M. Niu H. Guo C. J. (2017). Sensory neurons co-opt classical immune signaling pathways to mediate chronic itch. Cell 171, 217–228 e213. 10.1016/j.cell.2017.08.006 28890086 PMC5658016

[B122] Oettgen H. C. Geha R. S. (2001). IgE regulation and roles in asthma pathogenesis. J. Allergy Clin. Immunol. 107, 429–440. 10.1067/mai.2001.113759 11240941

[B123] Ortega H. G. Liu M. C. Pavord I. D. Brusselle G. G. FitzGerald J. M. Chetta A. (2014). Mepolizumab treatment in patients with severe eosinophilic asthma. N. Engl. J. Med. 371, 1198–1207. 10.1056/NEJMoa1403290 25199059

[B124] Panettieri R. A. Sjobring U. Peterffy A. Wessman P. Bowen K. Piper E. (2018). Tralokinumab for severe, uncontrolled asthma (STRATOS 1 and STRATOS 2): two randomised, double-blind, placebo-controlled, phase 3 clinical trials. Lancet Respir. Med. 6, 511–525. 10.1016/S2213-2600(18)30184-X 29792288

[B125] Patella V. Florio G. Petraroli A. Marone G. (2000). HIV-1 gp120 induces L-4 and IL-13 release from human Fc epsilon RI+ cells through interaction with he VH3 region of IgE. J. Immunol. 164, 589–595. 10.4049/jimmunol.164.2.589 10623799

[B126] Pearlman A. N. Chandra R. K. Chang D. Conley D. B. Tripathi-Peters A. Grammer L. (2009). Relationships between severity of chronic hinosinusitis and nasal polyposis, asthma, and atopy. Am. J. Rhinol. Allergy 23, 45–148. 10.2500/ajra.2009.23.3284 PMC3747516 19401038

[B127] Pepper A. N. Renz H. Casale T. B. Garn H. (2017). Biologic Therapy and ovel Molecular Targets of Severe Asthma. J. Allergy Clin. Immunol. Pract. 5, 909–916. 10.1016/j.jaip.2017.04.038 28689841

[B128] Pham T. H. Bak Y. Oh J. W. Hong J. Lee S. Hong J. T. (2019). Inhibition of IL-13 and IL-13Ralpha2 Expression by IL-32theta in Human Monocytic Cells Requires PKCdelta and STAT3 Association. Int. J. Mol. Sci. 20, E1949. 10.3390/ijms20081949 31010051 PMC6514684

[B129] Piper E. Brightling C. Niven R. Oh C. Faggioni R. Poon K. (2013). A hase II placebo-controlled study of tralokinumab in moderate-to-severe asthma. Respir. J. 41, 330–338. 10.1183/09031936.00223411 PMC3561510 22743678

[B130] Pope S. M. Brandt E. B. Mishra A. Hogan S. P. Zimmermann N. Matthaei K. I. (2001). IL-13 induces eosinophil recruitment into the lung by an L-5- and eotaxin-dependent mechanism. J. Allergy Clin. Immunol. 108, 594–601. 10.1067/mai.2001.118600 11590387

[B131] Poposki J. A. Klingler A. I. Tan B. K. Soroosh P. Banie H. Lewis G. (2017). Group 2 innate lymphoid cells are elevated and activated in chronic rhinosinusitis with nasal polyps. Immun. Inflammation Dis. 5, 233–243. 10.1002/iid3.161 PMC5569375 28474861

[B132] Prieto J. Lensmar C. Roquet A. van der Ploeg I. Gigliotti D. Eklund A. (2000). Increased interleukin-13 mRNA expression in bronchoalveolar lavage ells of atopic patients with mild asthma after repeated low-dose allergen rovocations. Respir. Med. 94, 806–814. 10.1053/rmed.2000.0826 10955758

[B133] Punnonen J. Aversa G. Cocks B. G. McKenzie A. N. Menon S. Zurawski G. (1993). Interleukin 13 induces interleukin 4-independent IgG4 and IgE synthesis and CD23 expression by human B cells. Proc. Natl. Acad. Sci. U. S. A. 90, 730–3734. 10.1073/pnas.90.8.3730 8097323 PMC46375

[B134] Rabe K. F. Nair P. Brusselle G. Maspero J. F. Castro M. Sher L. (2018). Efficacy and safety of dupilumab in glucocorticoid-dependent severe asthma. N. Engl. J. Med. 378, 2475–2485. 10.1056/NEJMoa1804093 29782224

[B135] Ragab A. Clement P. Vincken W. (2004). Objective assessment of lower airway involvement in chronic rhinosinusitis. Am. J. Rhinol. 18, 15–21. 10.1177/194589240401800105 15035566

[B136] Ramirez-Carrozzi V. Sambandam A. Zhou M. Yan D. Kang J. Wu X. (2017). Combined blockade of the IL-13 and IL-33 pathways leads to a greater inhibition of type 2 inflammation over inhibition of either pathway alone. J. Allergy Clin. Immunol. 139, 705–708 e706. 10.1016/j.jaci.2016.08.026 27697499

[B137] Redrup A. C. Howard B. P. MacGlashan D. W. Kagey-Sobotka A. Lichtenstein L. M. Schroeder J. T. (1998). Differential regulation of IL-4 and IL-13 ecretion by human basophils: their relationship to histamine release in mixed eukocyte cultures. J. Immunol. 160, 1957–1964. 9469459

[B138] Ricciardolo F. L. M. Sorbello V. Folino A. Gallo F. Massaglia G. M. avata G. (2017). Identification of IL-17F/frequent exacerbator endotype in sthma. J. Allergy Clin. Immunol. 140, 395–406. 10.1016/j.jaci.2016.10.034 27931975

[B139] Robinson D. Humbert M. Buhl R. Cruz A. A. Inoue H. Korom S. (2017). Revisiting Type 2-high and Type 2-low airway inflammation in asthma: current knowledge and therapeutic implications. Clin. Exp. Allergy 47, 161–175. 10.1111/cea.12880 28036144

[B140] Rosenberg H. F. Phipps S. Foster P. S. (2007). Eosinophil trafficking in allergy and asthma. J. Allergy Clin. Immunol. 119, 1303–1310. 10.1016/j.jaci.2007.03.048 17481712

[B141] Russell R. J. Chachi L. FitzGerald J. M. Backer V. Olivenstein R. itlestad I. L. (2018). Effect of tralokinumab, an interleukin-13 neutralising monoclonal antibody, on eosinophilic airway inflammation in uncontrolled moderate-to-severe asthma (MESOS): a multicentre, double-blind, randomised, placebo-controlled phase 2 trial. Lancet Respir. Med. 6, 499–510. 10.1016/S2213-2600(18)30201-7 29793857

[B142] Saha S. K. Berry M. A. Parker D. Siddiqui S. Morgan A. May R. (2008). Increased sputum and bronchial biopsy IL-13 expression in severe asthma. Allergy Clin. Immunol. 121, 685–691. 10.1016/j.jaci.2008.01.005 PMC3992379 18328894

[B143] Samitas K. Delimpoura V. Zervas E. Gaga M. (2015). Anti-IgE treatment, airway inflammation and remodelling in severe allergic asthma: current nowledge and future perspectives. Eur. Respir. Rev. 24, 594–601. 10.1183/16000617.00001715 26621973 PMC9487615

[B144] Samitas K. Zervas E. Gaga M. (2017). T2-low asthma: current approach to diagnosis and therapy. Curr. Opin. Pulm. Med. 23, 48–55. 10.1097/MCP.0000000000000342 27798418

[B145] Schmid-Grendelmeier P. Altznauer F. Fischer B. Bizer C. Straumann A. Menz G. (2002). Eosinophils express functional IL-13 in eosinophilic nflammatory diseases. J. Immunol. 169, 1021–1027. 10.4049/jimmunol.169.2.1021 12097410

[B146] Sekiya K. Nakatani E. Fukutomi Y. Kaneda H. Iikura M. Yoshida M. (2016). Severe or life-threatening asthma exacerbation: patient heterogeneity dentified by cluster analysis. Clin. Exp. Allergy 46, 1043–1055. 10.1111/cea.12738 27041475

[B147] Settipane G. A. Chafee F. H. (1977). Nasal polyps in asthma and rhinitis. A eview of 6,037 patients. J. Allergy Clin. Immunol. 59, 17–21. 10.1016/0091-749(77)90171-3 833373

[B148] Shimokawa C. Kanaya T. Hachisuka M. Ishiwata K. Hisaeda H. Kurashima Y. (2017). Mast cells are crucial for induction of group 2 innate ymphoid cells and clearance of helminth infections. Immunity 46, 863–874 864. 10.1016/j.immuni.2017.04.017 28514691

[B149] Singh D. Kane B. Molfino N. A. Faggioni R. Roskos L. Woodcock A. (2010). A phase 1 study evaluating the pharmacokinetics, safety and tolerability of repeat dosing with a human IL-13 antibody (CAT-354) in subjects with asthma. MC Pulm. Med. 10, 3. 10.1186/1471-2466-10-3 PMC2820465 20064211

[B150] Steinke J. W. (2004). Anti-interleukin-4 therapy. Immunol. Allergy Clin. North Am. 24, 599–614. 10.1016/j.iac.2004.06.008 15474861

[B151] Terl M. Sedlak V. Cap P. Dvorakova R. Kasak V. Koci T. (2017). Asthma management: a new phenotype-based approach using presence of osinophilia and allergy. Allergy 72, 1279–1287. 10.1111/all.13165 28328094

[B152] Truyen E. Coteur L. Dilissen E. Overbergh L. Dupont L. J. Ceuppens J. L. (2006). Evaluation of airway inflammation by quantitative Th1/Th2 cytokine mRNA measurement in sputum of asthma patients. Thorax 61, 202–208. 10.1136/thx.2005.052399 16449261 PMC2080739

[B153] Varricchi G. Senna G. Loffredo S. Bagnasco D. Ferrando M. Anonica G. W. (2017). Reslizumab and Eosinophilic Asthma: One Step Closer to recision Medicine? Front. Immunol. 8, 242. 10.3389/fimmu.2017.00242 28344579 PMC5344894

[B154] Varricchi G. Galdiero M. R. Loffredo S. Lucarini V. Marone G. Mattei F. (2018a). Eosinophils: the unsung heroes in cancer? Oncoimmunology 7, 1393134. 10.1080/2162402X.2017.1393134 PMC5749653 29308325

[B155] Varricchi G. Loffredo S. Galdiero M. R. Marone G. Cristinziano L. Granata F. (2018b). Innate effector cells in angiogenesis and lymphangiogenesis. Curr. Opin. Immunol. 53, 152–160. 10.1016/j.coi.2018.05.002 29778674

[B156] Varricchi G. Pecoraro A. Marone G. Criscuolo G. Spadaro G. enovese A. (2018c). Thymic stromal lymphopoietin isoforms, inflammatory isorders, and cancer. Front. Immunol. 9, 1595. 10.3389/fimmu.2018.01595 30057581 PMC6053489

[B157] Varricchi G. Raap U. Rivellese F. Marone G. Gibbs B. F. (2018d). Human mast cells and basophils-How are they similar how are they different? Immunol. Rev. 282, 8–34. 10.1111/imr.12627 29431214

[B158] Varricchi G. Rossi F. W. Galdiero M. R. Granata F. Criscuolo G. Spadaro G. (2019). Physiological roles of mast cells: collegium internationale allergologicum update 2019. Int. Arch. Allergy Immunol. 179, 247–261. 10.1159/000500088 31137021

[B159] Venkataramani S. Low S. Weigle B. Dutcher D. Jerath K. Menzenski M. (2018). Design and characterization of Zweimab and Doppelmab, high ffinity dual antagonistic anti-TSLP/IL13 bispecific antibodies. Biochem. Biophys. Res. Commun. 504, 19–24. 10.1016/j.bbrc.2018.08.064 30126632

[B160] Verma M. Liu S. Michalec L. Sripada A. Gorska M. M. Alam R. (2018). Experimental asthma persists in IL-33 receptor knockout mice because of the emergence of thymic stromal lymphopoietin-driven IL-9(+) and IL-13(+) type 2 innate lymphoid cell subpopulations. J. Allergy Clin. Immunol. 142, 793–803 e798. 10.1016/j.jaci.2017.10.020 29132961 PMC5945345

[B161] Wallrapp A. Riesenfeld S. J. Burkett P. R. Kuchroo V. K. (2018). Type 2 innate lymphoid cells in the induction and resolution of tissue inflammation. Immunol. Rev. 286, 53–73. 10.1111/imr.12702 30294962 PMC7370855

[B162] Walter D. M. McIntire J. J. Berry G. McKenzie A. N. Donaldson D. D. DeKruyff R. H. (2001). Critical role for IL-13 in the development of allergen-induced airway hyperreactivity. J. Immunol. 167, 4668–4675. 10.4049/jimmunol.167.8.4668 11591797

[B163] Wang I. M. Lin H. Goldman S. J. Kobayashi M. (2004). STAT-1 is activated by IL-4 and IL-13 in multiple cell types. Mol. Immunol. 41, 873–884. 10.1016/j.molimm.2004.04.027 15261459

[B164] Webb D. C. McKenzie A. N. Koskinen A. M. Yang M. Mattes J. Foster P. S. (2000). Integrated signals between IL-13, IL-4, and IL-5 regulate airways hyperreactivity. J. Immunol. 165, 108–113. 10.4049/jimmunol.165.1.108 10861042

[B165] Wenzel S. Wilbraham D. Fuller R. Getz E. B. Longphre M. (2007). Effect of an interleukin-4 variant on late phase asthmatic response to allergen challenge in asthmatic patients: results of two phase 2a studies. Lancet 370, 1422–1431. 10.1016/S0140-6736(07)61600-6 17950857

[B166] Wenzel S. E. (2012). Asthma phenotypes: the evolution from clinical to molecular approaches. Nat. Med. 18, 716–725. 10.1038/nm.2678 22561835

[B167] Wills-Karp M. Luyimbazi J. Xu X. Schofield B. Neben T. Y. Karp C. L. (1998). Interleukin-13: central mediator of allergic asthma. Science 282, 2258–2261. 10.1126/science.282.5397.2258 9856949

[B168] Wills-Karp M. (2001). IL-12/IL-13 axis in allergic asthma. J. Allergy Clin. Immunol. 107, 9–18. 10.1067/mai.2001.112265 11149983

[B169] Wood N. Whitters M. J. Jacobson B. A. Witek J. Sypek J. P. Kasaian M. (2003). Enhanced interleukin (IL)-13 responses in mice lacking IL-13 receptor alpha 2. J. Exp. Med. 197, 703–709. 10.1084/jem.20020906 12642602 PMC2193851

[B170] Wynn T. A. (2015). Type 2 cytokines: mechanisms and therapeutic strategies. Nat. Rev. Immunol. 15, 271–282. 10.1038/nri3831 25882242

[B171] Xu Y. Li J. Ding Z. Li B. Yu Z. Tan W. (2017). Association between IL-13 +1923C/T polymorphism and asthma risk: a meta-analysis based on 26 case-control studies. Biosci. Rep. 37, BSR20160505. 10.1042/BSR20160505 28057889 PMC5270317

[B172] Zhang F. Q. Han X. P. Zhang F. Ma X. Xiang D. Yang X. M. (2017). Therapeutic efficacy of a co-blockade of IL-13 and IL-25 on airway inflammation and remodeling in a mouse model of asthma. Int. Immunopharmacol. 46, 133–140. 10.1016/j.intimp.2017.03.005 28282577

[B173] Zhang Y. Cheng J. Li Y. He R. Pan P. Su X. (2019). The safety and efficacy of anti-IL-13 treatment with Tralokinumab (CAT-354) in moderate to severe asthma: a systematic review and meta-analysis. J. Allergy Clin. Immunol. Pract. 2661–2671. 10.1016/j.jaip.2019.05.030 31152798

[B174] Zhu Z. Homer R. J. Wang Z. Chen Q. Geba G. P. Wang J. (1999). Pulmonary expression of interleukin-13 causes inflammation, mucus hypersecretion, subepithelial fibrosis, physiologic abnormalities, and eotaxin production. J. Clin. Invest. 103, 779–788. 10.1172/JCI5909 10079098 PMC408149

